# Prevalence, awareness, control and determinants of hypertension among primary health care professional nurses in Eastern Cape, South Africa

**DOI:** 10.4102/phcfm.v10i1.1758

**Published:** 2018-11-22

**Authors:** Sizeka Monakali, Daniel Ter Goon, Eunice Seekoe, Eyitayo O. Owolabi

**Affiliations:** 1 Department of Nursing Science, Faculty of Health Sciences, University of Fort Hare, South Africa

## Abstract

**Background:**

Nurses in primary health care settings are key stakeholders in the diagnosis and management of hypertensive patients. Unfortunately, the working conditions of nurses predispose them to stress, long hours of work, shift duties and unhealthy diets, which are drivers of hypertension. Yet nurses are often overlooked in health screening exercises, primarily because they are assumed to be informed and ‘healthy’.

**Aim:**

This study examined the prevalence, awareness, control and determinants of hypertension among professional primary health care nurses in the Eastern Cape Province of South Africa.

**Setting:**

This was a cross-sectional survey of 203 professional nurses working at 41 primary health care facilities of the Eastern Cape Province.

**Methods:**

A modified WHO STEPwise questionnaire was used for data collection during face-to-face interviews. The information obtained included demographic information, behavioural lifestyles, anthropometric and blood pressure (BP) measurements. Hypertension is defined as an average of two BP ≥ 140/90 mmHg or self-reported history of antihypertensive medication use.

**Results:**

The prevalence of hypertension was 52%. Of this, 41% were unaware of their hypertension status. Of those who were aware and on treatment, only 38.1% had a controlled blood pressure. After adjusting for confounders (for physical activity, dietary practices, parity, income and alcohol use), only age and duration of practice were independent predictors of hypertension among the study population.

**Conclusion:**

There is a high prevalence of hypertension among the study participants. There is an unexpected low rate of awareness and suboptimal control of blood pressure among the participants. Age is the significant predictor of hypertension among professional nurses in Eastern Cape Province, South Africa. There is an urgent need for the implementation of an effective workplace health programme for nurses in the province.

## Introduction

Hypertension is a global public health concern and a marker of complex cardiovascular malady; furthermore, it is an independent and preventable risk factor for morbidity and mortality. Twenty-two percent of the world’s adult population currently suffers from hypertension.^1^ Contrary to earlier reports of hypertension being a disease predominantly affecting the rich, it is now diagnosed even among the poor.^2^ Currently, more than 80% of hypertension cases are seen in low- and middle-income countries with variance across different countries, gender, age and socio-economic categories.^2,3^ South Africa, like other African countries, is also challenged by hypertension; this is in addition to South Africa’s burden of communicable diseases and maternal mortality.^4^

Worryingly, the burden of hypertension in Africa is complicated by under-diagnosis, poor treatment and control, as a result of its asymptomatic nature.^5^ This is however a risky adventure as undiagnosed and uncontrolled hypertension potentially increases the chance of developing target organ damage and other life-threatening conditions, whereas early diagnosis affords the opportunity for prompt intervention.^5^ Several factors are responsible for the development of hypertension. Among these are behavioural factors such as unhealthy diets, obesity, physical inactivity, harmful alcohol and tobacco use. However, work-related factors such as stress and sedentary behaviour have also been implicated in the aetiology of hypertension.

The focus of health managers, health care workers and even researchers regarding hypertension is constantly on the patients and the entire population. Blood pressure (BP) measurement is one of the commonly observed vital signs during clinical assessment. Even if other assessments are not done, BP measurement is inevitable in almost all clinical visits. However, it is unclear whether health workers, particularly the nurses involved in clinical assessments of patients, do care enough to assess their own health. Health workers, particularly nurses, are an essential population group whose service is invaluable in fostering better health among the populace. As one of the conveyors of health information, nurses are health role models for patients and communities. Unfortunately, the working conditions of nurses predisposes them to stress, long hours of work, shift duties and unhealthy diets, which are drivers of hypertension.^6,7,8^ Yet nurses are often not included in health screening exercises, probably because they are presumed healthy. Primary health care (PHC) nurses are usually the first contact health personnel for a large part of the populace. Although PHC nurses in South Africa mostly do not run shift duties, they are exposed to long hours of sitting during consultations and seldom engage in physical activity. There is hardly any literature documenting the prevalence, awareness and treatment of hypertension among nurses in South Africa. In view of this, this study sought to determine the prevalence, awareness and control of hypertension among PHC nurses in the Eastern Cape, South Africa. Such information could ascertain the health levels of PHC professional nurses and could contribute towards the design of effective workplace health programmes and policies.

## Methods

### Study design

This was a workplace, cross-sectional, population study involving 203 PHC professional nurses conveniently selected across 41 PHC facilities in the Eastern Cape Province.

### Study setting

The Eastern Cape is one of the nine provinces of South Africa, with its capital in Bisho. It was created in 1994 from the Xhosa homelands of Transkei and Ciskei together with the eastern segment of the Cape Province. It is the traditional home of the Xhosa people. The province is made up of two metropolitan municipalities – Buffalo City Metropolitan Municipality (BCMM) and Nelson Mandela Metropolitan Municipality – and six districts. These districts are Amathole, Joe Gqabi, OR Tambo, Sarah Baartman, Chris Hani and Alfred Nzo.^9^

### Study population and sampling

**FIGURE 1:**
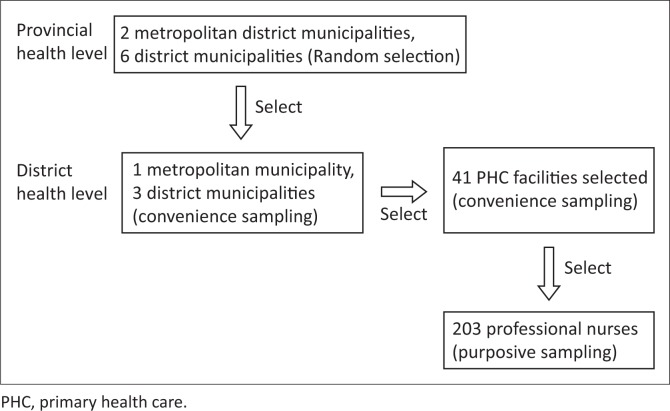
Flowchart showing study sampling.

Primary health care nurses in South Africa form the first contact with the community and were chosen as subjects among whom to ascertain the burden of hypertension. Of the six districts and two metropoles of the Eastern Cape Province, four were randomly selected, namely, BCMM, OR Tambo, Chris Hani and Sarah Baartman districts. In the randomly selected districts and municipalities, a convenience sampling method was used to select the PHC centres. There are approximately 880 nurses in the selected districts because of staff shortage, especially in rural districts like OR Tambo (OR Tambo = 250, Chris Hani = 230, Sarah Baartman = 100 and BCMM = 300). At a confidence level of 95%, a sample size of 268 nurses is required. However, only 203 were available and accessible (Figure 1). Only practising professional nurses aged 18 years and above were included in the study. The pregnant nurses were excluded as a result of effect on obtaining anthropometric measurements. The study took place between February to May of 2017.

### Eligibility criteria

Participants were included in the study if they were professional nurses, practising in PHC clinics, aged 18 years and above, available on duty during data collection and willing to participate in the study. Exclusion criteria included pregnancy or any form of debilitation that makes obtaining anthropometric measures difficult.

### Data collection

The modified WHO STEPwise questionnaire (which comprises three major items, namely demographic data, behavioural data and measurements) was the data collection tool. A pilot study was carried out among 20 nurses in the Amathole district, which was not included in the main study in order to establish the suitability of the instrument. Modifications and corrections were done afterwards.

Data for demographic and behavioural characteristics were obtained by self-reporting during face-to-face interviews. Demographic variables included items on gender, age, marital status, level of education, employment status, duration of practice and average monthly income. The following behavioural variables were obtained by self-reporting: smoking, alcohol use and physical activity. Smoking and alcohol use were assessed by self-reporting on the use of any tobacco product or alcoholic drink in the past 30 days. Participants’ levels of physical activity were obtained by self-reported engagement in moderate (*yes* or *no*) or vigorous (*yes* or *no*) intensity exercise.

### Measurements

Participants’ BP (systolic and diastolic) was measured in accordance with standard protocol^10^ using a validated Microlife BP A100 Plus model (Widnau, Switzerland). Two BP readings were done five minutes apart whilst the participants were sitting in an upright position. Hypertension was defined as an average of two systolic BP measurements of ≥ 140 mmHg or a diastolic BP of ≥ 90 mmHg, in accordance with the Eighth Joint National Committee guidelines.^11^

Anthropometric measurements followed the standard anthropometric methods of the International Society for the Advancement of Kinanthropometry.^12^ Body weight was measured in light clothes to the nearest 0.01 kg in a standing position using a SECA scale (SECA Co., North America) and height was measured to the nearest 0.1 m by stadiometer in a standing position with closed feet, without shoes. Waist circumference was measured at the level of the narrowest point between the lower costal border and the iliac crest at the end of a normal exhalation with the arms relaxed at the sides. The hip circumference was measured at the widest circumference of the buttocks with a non-extensible tape to the nearest 0.1 cm. All measurements were taken by a trained research assistant to ensure uniformity.

### Data analysis

Data were analysed using Statistical Package for the Social Sciences (SPSS) version 21 for Windows (SPSS Inc., Chicago, IL, United States). Data were expressed as mean values ± standard deviations (SD) for continuous variables. Frequencies (*n*) and percentages (%) were reported for categorical variables. Counts (frequency = *n*) and percentages (%) were reported for categorical variables. Percentages were compared using the chi-square test. The logistic regression model analysis adjusted for obesity, physical activity, dietary practices, parity, income and alcohol use. A *p*-value of < 0.05 was considered statistically significant.

## Results

The average age of the study participants was 45.17 (SD ± 11.26) years whilst the average duration of practice was 15.98 (SD ± 11.07) years. Almost half (49.8%) of the participants were married and 40.9% were single. The majority were black (96.1%), had a diploma certificate in nursing (65%) and earned more than R15 000.00 (60.6%) per month (Table 1).

**TABLE 1:** Socio-demographic characteristics of participants by gender.

Variables	All		Female		Male	
	*n*	%	*n*	%	*n*	%
**Age**						
21 years – 30 years	26	12.8	19	10.6	7	29.2
31 years – 40 years	50	24.6	41	22.9	9	37.5
41 years – 50 years	42	20.7	39	21.8	3	12.5
51 years – 60 years	74	36.5	69	38.5	5	20.8
61 years – 70 years	11	5.4	11	6.1	0	0.0
**Marital status**						
Single	83	40.9	71	39.7	12	50.0
Married	101	49.8	90	50.3	11	45.8
Divorced	9	4.4	9	5.0	0	0.0
Separated	1	0.5	1	0.6	0	0.0
Widow or widower	9	4.4	8	4.5	1	4.2
**Number of children**						
1	37	20.4	33	20.4	4	21.1
2	61	33.7	58	35.8	3	15.8
3	58	32.0	49	30.2	9	47.4
4	18	9.9	16	9.9	2	10.5
5	5	2.8	5	3.1	0	0.0
6	1	0.6	1	0.6	0	0.0
7	1	0.6	0	0.0	1	5.3
**Level of education**						
Diploma	132	65.0	117	65.4	15	62.5
Degree	67	33.0	58	32.4	9	37.5
Postgraduate diploma	3	1.5	3	1.7	0	0.0
Master’s	1	0.5	1	0.6	0	0.0
**Race**						
Black people	195	96.1	171	95.5	24	100.0
Mixed race people	8	3.9	8	4.5	0	0.0
**Duration of practice**						
1 year – 10 years	72	40.7	59	38.1	13	59.1
11 years – 20 years	41	23.2	37	23.9	4	18.2
21 years – 30 years	44	24.9	41	26.5	3	13.6
31 years and above	20	11.3	18	11.6	2	9.1
**Income**						
10 000 – 15 000	65	39.4	55	38.5	10	45.5
Above 15 000	100	60.6	88	61.5	12	54.5

**FIGURE 2:**
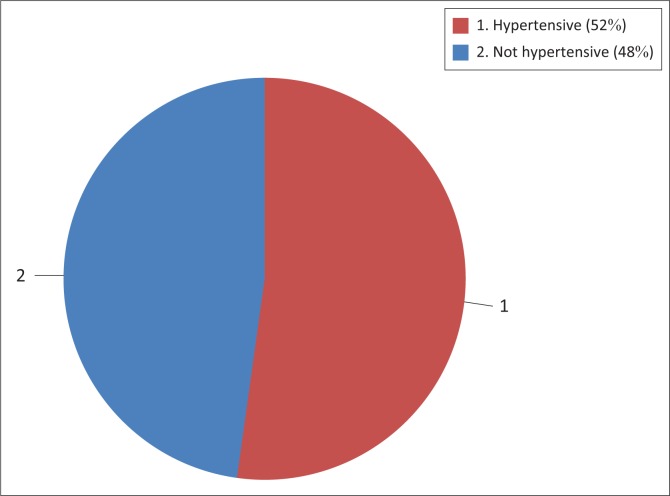
Prevalence of hypertension.

Of the participants, 52% were hypertensive (Figure 2).

Of those who were hypertensive, 41% were unaware, and of those who were aware and on treatment, only 38.1% were controlled. Table 2 showed that only age, alcohol use, parity, duration of practice, income and obesity were associated with hypertension among the study participants.

**TABLE 2:** Association of hypertension with demographic variables.

Variables	Hypertension		Not hypertensive		*p*
	*n*	%	*n*	%	
**Gender**	-	-	-	-	0.338
Male	14	58.3	10	41.7	-
Female	92	51.4	87	48.6	-
**Marital status**	-	-	-	-	0.234
Single	37	44.6	46	55.4	-
Married	59	58.4	42	41.6	-
Divorced	4	44.4	5	55.6	-
Separated	0	0.0	1	100.0	-
Widow or widower	6	66.7	3	33.3	-
**Number of children**	-	-	-	-	0.028
1	15	40.5	22	59.5	-
More than 1	86	59.7	58	40.3	-
**Level of education**	-	-	-	-	0.307
Diploma	74	56.1	58	43.9	-
Degree	30	44.8	37	55.2	-
Postgraduate diploma	2	66.7	1	33.3	-
Master’s	0	0.0	1	100.0	-
**Race**	-	-	-	-	0.589
Black people	102	52.3	93	47.7	-
Mixed race people	4	50.0	4	50.0	-
**Age**	-	-	-	-	< 0.001
21–30	2	7.6	24	92.3	-
31–40	19	38.0	31	62.0	-
41–50	17	40.5	25	59.5	-
51–60	58	78.4	16	21.6	-
61–70	10	90.9	1	9.1	-
**Duration of practice**	-	-	-	-	< 0.001
1–10 years	17	23.6	55	76.4	-
11–20 years	24	58.5	17	41.5	-
21–30 years	34	77.3	10	22.7	-
31 and above	18	90.0	2	10.0	-
**Income**	-	-	-	-	0.043
10 000–15 000	28	43.1	37	56.9	-
Above 15 000	58	58.0	42	42.0	-
**Alcohol use**	-	-	-	-	0.001
Yes	18	33.3	36	66.7	-
No	88	59.1	61	40.9	-
**Smoking**	-	-	-	-	0.242
Yes	7	41.2	10	58.8	-
No	99	53.2	87	46.8	-
**Level of activity**	-	-	-	-	0.580
Inactive	32	48.5	34	51.5	-
Less active	40	51.3	38	48.7	-
Active	34	57.6	25	42.2	-
**Obesity**	-	-	-	-	0.001
Yes	55	57.9	40	42.1	-
No	15	30.0	35	70.0	-

In the adjusted model, only age and duration of practice were the significant predictors of hypertension (Table 3).

**TABLE 3:** Multivariate logistic showing predictors of hypertension.

Variables	*β*	s.e.	Wald	OR	95% CI	*p*
**Duration of practice**	-	-	-	-	-	< 0.001
≤ 10 years (reference)	1.49	0.39	14.42	4.4	2.1–9.6	-
> 10 years	-	-	-	-	-	-
**Age**	-	-	-	-	-	0.002
≤ 35 years (reference)	1.89	0.60	9.8	6.6	2.0–21.7	-
> 35 years	-	-	-	-	-	-

Note: Most of the characteristics that showed significant association in the univariate analysis failed to demonstrate any association following the multivariate analysis.

*β*, beta coefficient; s.e., standard error, OR, odds ratio; CI, confidence interval.

## Discussion

Hypertension is a leading, preventable cause of morbidity and premature mortality. This study sought to determine the prevalence, awareness, control and determinants of hypertension among professional PHC nurses in the Eastern Cape Province. The prevalence rate of hypertension among the professional nurses was 52%. This prevalence is higher than the reported prevalence of hypertension among nurses in South Africa (20%),^7^ Brazil (32%)^8^ and health workers in Nigeria (20.1%).^13^ This finding is also higher than the documented hypertension prevalence among adults in the general population in South Africa,^14,15,16^ with prevalence ranging from 38.9% – 49.2%. This paradoxically means that the health of nurses who are caring for the health of the general populace has been compromised, as they too are patients who need to be cared for. A study assessing lifestyle behaviour among nurses in South Africa documented a high prevalence of unhealthy lifestyle behaviours among South African nurses.^5^ This finding, coupled with the work-related stress and long sitting hours that characterise the nursing profession and PHC practice might be responsible for the higher prevalence of hypertension found among the nurses, as reported in other studies.^6,16^ Furthermore, the mean age of the study participants might have contributed to the high prevalence. Nonetheless, the prevalence is high and this is a call for action by the provincial health managers, as a matter of urgency, to organise effective workplace health programmes targeting this very important group.

Forty-one percent of the hypertensive nurses were unaware of their hypertensive status. This is a cause for concern as some of the key stakeholders involved in reducing the burden of undiagnosed hypertension are not involved in the disease prevention measures for which they advocate. However, it is unclear whether this attitude can be attributed to work-related stress, which is highly documented among this group^7,17^, such as that they forget to even engage in health assessments, of which BP measurement is a prime priority. Worryingly, hypertension is asymptomatic, does not give warnings before creeping in and silently affects several organs in the body. This signifies the need to enforce workplace screening even among this group that supposedly has health knowledge and an awareness of the disease and its deleterious health effects. Periodic screening exercise could increase awareness and early detection of hypertension, which might be life-saving.

Of those participants who were aware of their hypertension status and were on treatment, only 38.1% had their BP well controlled. The rate is higher than the reported control rate of adults in Mthatha, South Africa (25.5%),^18^ and in Zimbabwe (32.8%).^19^ This study’s findings is comparable to the reported rate (38.9%) among adults in Buffalo City Metropole, South Africa.^16^ The findings are also comparable to a household survey that was conducted among South African adults (36.4%).^14^ Despite the scientific successes recorded in anti-hypersensitive drug discoveries, achieving treatment target remains a significant challenge in most developing countries. The nurses are not exempted from the burden of suboptimal control of hypertension. Intensified efforts towards better hypertension control in South Africa are vital.

Only age and the duration of practice were significant predictors of hypertension among the study population. The association between age and hypertension has been documented in several studies.^16,20,21^ Hypertension is inevitable among the elderly, a result of the changes in the body systems, including the cardiovascular system, as one ages. Changes such as arterial stiffness, inflammation and endothelial dysfunction that are associated with ageing^22,23^ account for the greater burden of hypertension found among the older population, including the nurses. This could also be a plausible reason for the documented higher odds among those with higher duration of practice, as they constitute those in the higher age range. Interventions for reducing the burden of hypertension among people of the older age group are crucial. There is a need for future studies examining this burden among health workers generally.

## Strengths and limitations

To our knowledge, this is the first study to assess the prevalence, awareness and control of hypertension among nurses in South Africa, and the findings of this study provide a snapshot of the burden of hypertension among this neglected population in health screenings. Furthermore, the coverage of several PHC clinics gives credence to this study. However, the cross-sectional nature and the small sample size are obvious limitations.

## Conclusion

There is a high prevalence of hypertension among the study group. Also, there is a low rate of awareness and control of hypertension among professional nurses in the Eastern Cape Province of South Africa. Only ageing and duration of practice were independent predictors of hypertension among the study population. There is an urgent need to implement effective and regular workplace health programmes for nurses in the Eastern Cape Province.
